# SimBac: simulation of whole bacterial genomes with homologous recombination

**DOI:** 10.1099/mgen.0.000044

**Published:** 2016-01-19

**Authors:** Thomas Brown, Xavier Didelot, Daniel J. Wilson, Nicola De Maio

**Affiliations:** ^1^​ Doctoral Training Centre, University of Oxford, Oxford, UK; ^2^​ Department of Infectious Disease Epidemiology, Imperial College, London, UK; ^3^​ Institute for Emerging Infections, Oxford Martin School, Oxford, UK; ^4^​ Nuffield Department of Medicine, University of Oxford, Oxford, UK; ^5^​ Wellcome Trust Centre for Human Genetics, University of Oxford, Oxford, UK

**Keywords:** bacterial evolution, bacterial genomics, bacterial recombination, coalescent simulation

## Abstract

Bacteria can exchange genetic material, or acquire genes found in the environment. This process, generally known as bacterial recombination, can have a strong impact on the evolution and phenotype of bacteria, for example causing the spread of antibiotic resistance across clades and species, but can also disrupt phylogenetic and transmission inferences. With the increasing affordability of whole genome sequencing, the need has emerged for an efficient simulator of bacterial evolution to test and compare methods for phylogenetic and population genetic inference, and for simulation-based estimation. We present SimBac, a whole-genome bacterial evolution simulator that is roughly two orders of magnitude faster than previous software and includes a more general model of bacterial evolution, allowing both within- and between-species homologous recombination. Since methods modelling bacterial recombination generally focus on only one of these two modes of recombination, the possibility to simulate both allows for a general and fair benchmarking. SimBac is available from https://github.com/tbrown91/SimBac and is distributed as open source under the terms of the GNU General Public Licence.

## Data Summary

SimBac, the software we developed to simulate genome-wide bacterial evolution, is distributed as open source under the terms of the GNU General Public Licence, and is available from GitHub (https://github.com/tbrown91/SimBac). A manual and examples of usage of SimBac are provided in the Supplementary Material.

## Impact Statement

Sequencing technologies are revolutionizing microbiology, allowing researchers to investigate with great detail the genetic information in bacteria. This increasingly overwhelming amount of information requires adequate, efficient computer methods to be processed in reasonable time. One of the most important tasks performed by computer methods is simulating data, as this provides a means for testing hypotheses and checking the performance of other methods in extracting valuable information from data. Previous software specifically developed for simulating bacterial evolution is limited in applicability, having been conceived for limited data and biological phenomena. We present SimBac, a new simulator of bacterial evolution that can generate data for thousands of bacterial genomes about 100 times faster than previous methods. SimBac also includes a very general model of bacterial evolution that accounts for the fact that bacteria can exchange genetic material with each other, not only within the same population, but also across species boundaries. Thanks to these advancements in SimBac it will be possible to efficiently test hypotheses and estimate parameters comparing real and simulated bacterial data, to test the accuracy of bacterial genomic methods, and to fairly compare methods that make different assumptions regarding bacterial evolution.

## Introduction

Whole-genome bacterial sequencing is rapidly gaining in popularity and replacing multilocus sequence typing (MLST) thanks to its fast and cost-effective provision of higher resolution genetic information (Didelot *et al.*, 2012; Wilson, 2012). Computational algorithms that use genomic data to infer epidemiological, phylogeographic, phylodynamic and evolutive patterns are generally hampered by recombination (e.g. Schierup & Hein, 2000; Posada & Crandall, 2002; Hedge & Wilson, 2014), and recent years have seen a surge of methods that measure, identify and account for bacterial homologous recombination (e.g. Didelot & Falush, 2007; Marttinen *et al.*, 2008, 2012; Didelot *et al.*, 2010; Croucher *et al.*, 2015; Didelot & Wilson, 2015).

Assessing and comparing the performance of different methods is complicated by the use of different models of recombination, in particular within-species recombination leading to phylogenetically discordant sites (e.g. Didelot *et al.*, 2010) or between-species recombination leading to accumulation of substitutions on specific branches and genomic intervals (e.g. Didelot & Falush, 2007). Simulators of bacterial evolution are routinely used for parameter inference and hypothesis testing (Fearnhead *et al.*, 2005; Fraser *et al.*, 2005) and for method testing and comparison (Falush *et al.*, 2006; Didelot & Falush, 2007; Turner *et al.*, 2007, Buckee *et al.*, 2008; Wilson *et al.*, 2009; Hedge & Wilson, 2014), but simulation software and models used are generally targeted to the specific model of evolution implemented in the methods considered. One of the reasons for this is the lack of general and efficient simulators of bacterial evolution.

Coalescent simulators of eukaryotic evolution usually focus on crossover recombination (see e.g. Arenas & Posada, 2007, 2010, 2014), while bacterial recombination is generally modelled as gene conversion, meaning that in a recombination event only a small fragment of DNA is imported from a donor, whereas most of the genetic material is inherited from the recipient. Many fast and approximate simulation methods (e.g. Marjoram & Wall, 2006; Excoffier & Foll, 2011) cannot be applied to bacterial recombination because the approximations used do not generate the expected long genomic distance correlations in bacterial local trees. Other similar approximate methods are only adequate for low bacterial recombination rates (e.g. Chen *et al.*, 2009; Wang *et al.*, 2014). Many forward-in-time simulation methods (e.g. Chadeau-Hyam *et al.*, 2008; Dalquen *et al.*, 2012) or discrete generation coalescent methods (Excoffier *et al.*, 2000; Laval & Excoffier, 2004) can allow gene conversion, but are generally too slow for simulating whole-genome evolution of large samples or populations.

An exact and fast method to simulate gene conversion is the coalescent model of Wiuf & Hein (2000) included in ms (Hudson, 2002) and its extensions (Mailund *et al.*, 2005; Hellenthal & Stephens, 2007; Ramos-Onsins & Mitchell-Olds 2007). Recently, this model has been implemented in simulation software specific for bacterial evolution, SimMLST (Didelot *et al.*, 2009).

SimMLST is optimized for MLST data which requires to simulate several short distant loci, and, similarly to ms, only simulates within-species bacterial recombination. For these reasons, these methods are not generally suited for large, genome-wide bacterial simulation studies or for testing different models and assumptions of recombination.

Here we present SimBac, a new method for simulating bacterial evolution. SimBac implements an efficient coalescent-based algorithm for simulating genome-wide bacterial evolution, and includes a new and more general model of bacterial recombination that extends the classical within-species recombination (Didelot *et al.*, 2009) by allowing the user to specify any degree of recombination between species.

## Theory and Implementation

We simulate evolution backward in time under the standard coalescent model with gene conversion, and generate an ancestral recombination graph (ARG; see Wiuf & Hein, 2000). Within-species recombination events are modelled as a copy-pasting of a small fragment of DNA from the donor lineage sequence into the recipient.

**Fig. 1:**
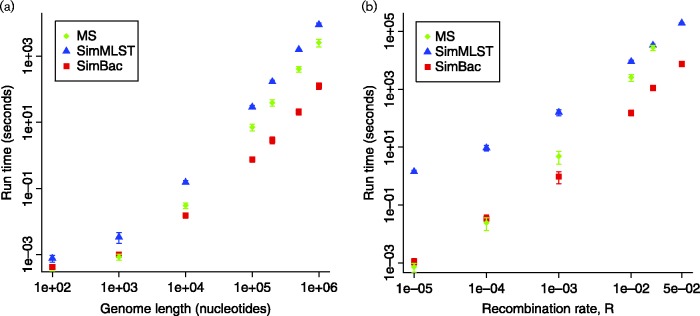
Comparison of run-time of SimMLST, ms and SimBac. Only gene conversion (no crossover) is simulated in ms, to model bacterial evolution. (a) Mean time to simulate the ARG for a fixed recombination rate *R* = 0.01 and genome length from 100 bp to 1 Mbp. (b) Mean time to simulate the ARG for a fixed genome length of 1 Mbp and recombination rate increasing from *R* = 0 to *R* = 0.05. One hundred simulations were performed for each dot, except for SimMLST at *R* = 0.02 and *R* = 0.05, and ms at *R* = 0.02, where 10 simulations were performed due to the elevated computational demand. ms was not run at *R* = 0.05 because a single run required >4 days. Error bars show ± 1 sd.

The computational efficiency of SimBac derives from algorithmic improvements over previous software. First, instead of rejection sampling of recombination events as described by Didelot *et al.* (2009), we developed an analytical solution that only samples recombination events effectively altering ancestral material of lineages (details of the methods are available in the online Supplementary Material). Second, we represent ancestral material with a more efficient data structure. These new features allow about 100-fold faster simulation of bacterial genome-wide evolution compared with SimMLST (see Fig. 1). Also, our method generally outperforms ms (Hudson, 2002) when many recombination (or equivalently gene conversion) events are expected.

**Fig. 2:**
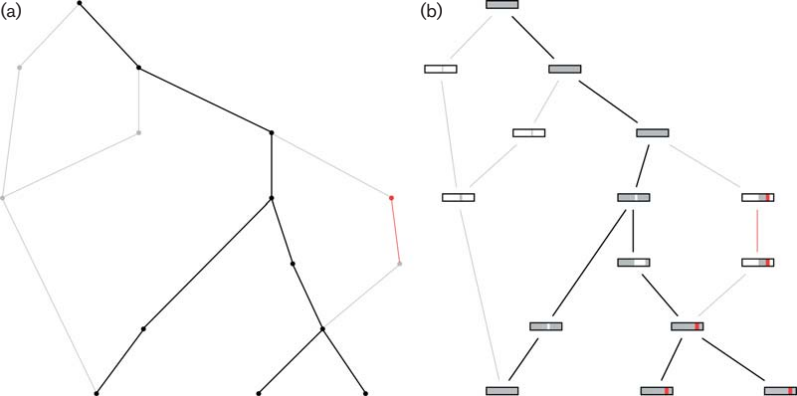
Examples of ancestral recombination graphs (ARGs) generated and plotted by SimBac. Branches represent ARG lineages, and time is considered to go backward from the bottom to the top of the tree. Branch merges (from bottom to top) represent coalescent events, while branch splits represent recombination events. (a) Example ARG with the clonal frame lineages marked in black, the non-clonal lineages in grey, and a recombination event involving an external species marked in red. (b) Same ARG as before, but with ancestral material of each lineage represented as a rectangle in the corresponding node. Each coloured vertical bar inside each rectangle represent a genomic segment. Genomic segments that are present in the ancestral material are coloured in grey, those absent are in white, and those imported from an external species are in red.

Our software also provides the possibility to simulate a circular or linear genome, and entire or fragmented bacterial genome, and offers a recombination model that allows a mixture of between- and within-species recombination. Within-species recombination is modelled as the coalescent with gene conversion (Wiuf & Hein, 2000; Didelot *et al.*, 2009) with fragment lengths distributed geometrically with mean δ, and with all sites having the same per-site recombination initiation rate *R* (scaled by the effective population size). As the coalescent process is simulated backward in time, any extant lineage can be the recipient of a recombining interval from a donor lineage, which is then added to the other extant lineages. In such a case, the recombining interval becomes part of the genome of the new donor lineage (see Fig. 2b). Every site of the genome of every extant lineage becomes the start of a recombining interval at the same rate *R*.

Between-species recombination is modelled as a separate process backward in time with a specific scaled per-site recombination initiation rate *R*_e_ and a specific distribution of imported fragment lengths (geometric with mean δ_e_). When a between-species recombination event occurs at a recipient lineage and interval, the donor lineage is not tracked back in time as for within-species recombination, but instead substitutions are introduced into the recombining interval, similar to the model in ClonalFrame (Didelot & Falush, 2007). Therefore, we do not simulate species evolution as described by Arenas & Posada (2014), but rather assume that each recombining segment is donated by a different lineage within a given divergence range.

However, differently from ClonalFrame, the donor sequence is obtained by adding a random amount of divergence [uniformly sampled within the interval (*D*_1_, *D*_2_), specified by the user] into the corresponding homologous sequence from the root of the ARG. This model accounts for the excess of substitutions caused by between-species recombination as in ClonalFrame, but at the same time also generates the homoplasies that are expected if the recipient lineage does not lead to the root of the local tree. More details on the methods of simulation and a summary of the algorithm are provided in the online Supplementary Material.

**Fig. 3:**
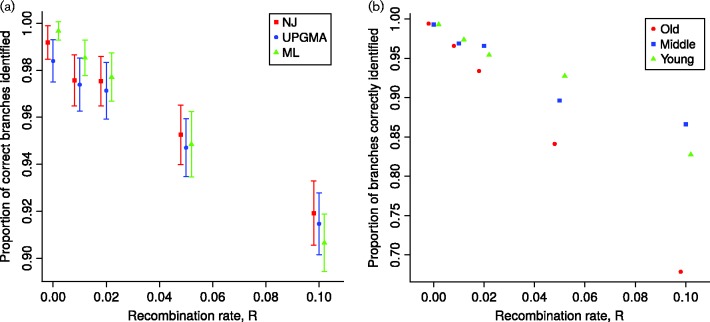
Accuracy of clonal frame estimation from recombining bacterial genomes. The *x*-axis shows the recombination rate *R* under which simulations are performed. The *y*-axis shows the accuracy of inference, as the proportion of branches correctly estimated using the Robinson–Foulds metric (Robinson & Foulds, 1981). Ten independent replicates are used for *R* = 0.1 and 100 in all other cases. Genomes are 1 Mbp long and the scaled mutation rate is fixed at 0.01. (a) Accuracy of three phylogenetic methods: neighbour-joining (NJ), unweighted pair group method with arithmetic mean (UPGMA) and maximum-likelihood (ML). Error bars represent ± 1 sd. (b) Clonal frame branches were separated into three age categories: young, middle-aged and old (respectively with a distance between the branch mid-point and the root of more than 2.09, between 1.32 and 2.09, and less than 1.32 *N*_*e*_ generations). The ML accuracy for each age category is plotted separately in different colours.

To showcase the possible applications of our software, we extend the investigation of phylogenetic inference accuracy by Hedge & Wilson (2014). The authors investigated the effect of low bacterial recombination rates (up to a scaled per-site rate of *R* = 0.01) on the inference of clonal frame. Using SimBac, we are able to simulate higher recombination rates (up to *R* = 0.1) in reasonable time, and we show that for highly recombining bacteria, and in particular for older phylogenetic branches, the probability of reconstructing the phylogenetic topology is reduced further to around 91 % (Fig. 3).

## Conclusion

Simulation of genome evolution is important as it allows inference of parameters from data and testing of evolutionary hypotheses, and because it is routinely used to benchmark and compare different microbial genomic analysis methods. We present SimBac, a new method for simulating genome-wide bacterial evolution implemented and distributed as open source software (*https://github.com/tbrown91/SimBac*). Our model of bacterial recombination is more general than those used by most methods in the field, in that it can describe any mixture of within-species and between-species recombination, and as such, it can fit the assumptions of most methods, or it can provide a more realistic background for comparing methods with different hypotheses.

Also, our efficient implementation achieves an approximately 100-fold increase in computational efficiency over previous similar efforts, allowing inference and benchmarking over considerably larger datasets. For example, 1000 1 Mbp genomes with *R = 0.01* can be generated in about 6 min. SimBac can generate a wide range of possible outputs: sequence alignments, ARGs graphics (see Fig. 2), clonal frames, local genealogies and lists of recombination events. Although only a Jukes & Cantor substitution model (Jukes & Cantor 1969) is presently included in SimBac, in practice this is not a restriction because the local genealogies can be used to generate alignments under a vast choice of nucleotide and codon substitution models using, for example, SeqGen (Rambaut & Grassly, 1997) or INDELible (Fletcher & Yang, 2009) (see Arenas, 2013).

Although SimBac generalizes the applicability of SimMLST, it currently lacks the wide set of options of some simulators of evolution, in particular of forward simulators that allow very general demographic, speciation, selection, migration and rate variation patterns (e.g. Chadeau-Hyam *et al.*, 2008; Dalquen *et al.*, 2012). In fact, many of these features present considerable methodological hurdles in being incorporated in computationally efficient coalescent simulators.

Yet, future extensions of our method could consist of the inclusion of distributive conjugal transfer (Gray *et al.*, 2013), of non-homogeneous genomic rates of recombination (see e.g. Everitt *et al.*, 2014; Arenas & Posada, 2014), or of demographic events and population structure (Arenas & Posada, 2007, 2014).

## Supplementary Data

274 Supplementary Data
