# Supplementary material for: SimBac: simulation of whole bacterial genomes with homologous recombination
Source: Microb Genom. 2016 Jan 19;2(1):e000044. doi: 10.1099/mgen.0.000044 (PMC5049688; doi:10.1099/mgen.0.000044)
Supplement: Supplementary file 1 — Supplementary Data [file mgen-02-44-s001.pdf]

SimBac: simulation of whole bacterial genomes with homologous recombination

Supplementary Information

Thomas Brown [1], Xavier Didelot [2], Daniel J. Wilson [3,4,5] and Nicola De Maio [3,4,*]

1 Doctoral Training Centre, University of Oxford, Oxford, United Kingdom

2 Department of Infectious Disease Epidemiology, Imperial College, London, United Kingdom

3 Institute for Emerging Infections, Oxford Martin School, Oxford, United Kingdom

4 Nuffield Department of Medicine, University of Oxford, Oxford, United Kingdom

5 Wellcome Trust Centre for Human Genetics, University of Oxford, Oxford, United Kingdom

*** E-mail: nicola.demaio@ndm.ox.ac.uk**

User Manual

SimBac jointly simulates bacterial genomes with the clonal genealogy under a coalescent model with recombination. Such simulations can be used to test phylogenetic analysis of real data sets.

Running from the command line

SimBac can be run from the command line using a combination of the following options. Passing no arguments to SimBac will display the possible arguments.

-N NUM Sets the number of isolates (default is 100)

-T NUM Sets the value of θ, the site-specific mutation rate, between 0 and 1 (default is 0.01)

-m NUM Sets the lower bound of site-mutation (divergence) in a region of external recombination, between 0 and 1 (default is 0)

-M NUM Sets the upper bound of site-mutation (divergence) in a region of external recombination, between 0 and 1 (default is 0)

-R NUM Sets the per-site rate of internal (within species) recombination, R_i, (default is 0.01)

-r NUM Sets the per-site rate of external (between species) recombination, R_e, (default is 0)

-D NUM Sets the average length of an internal recombinant interval, δ_i (default is 500)

-e NUM Sets the average length of an external recombinant interval, δ_e (default is 500)

-B NUM,. . .,NUM Sets the number and lengths of fragments of genetic material (default is 10000)

-G NUM,. . .,NUM Sets the size of gaps between each fragment, must be the same number of gaps as there are numbers of genetic fragments (default is 0,. . .,0)

-s NUM Use given seed to initiate random number generation

-o FILE Name of file to write generated sequences (FASTA format)

-c FILE Name of file to write clonal genealogy (Newick format)

-l FILE Name of file to write local trees (Newick format)

-b FILE Name of file to write log of internal recombination breaks

-f FILE Name of file to write log of external recombination breaks

-d FILE Name of file to export ancestral recombination graph (DOT file)

-a Include ancestral material in the DOT graph

Output format

SimBac produces the following output files:

- FASTA file of simulated sequences. If more than one fragment of genetic information is specified, the output is in the eXtended Multi-Fasta Alignment (XMFA) format. In this situation the simulated gene fragments are separated with an '=' sign.
- The clonal genealogy in Newick format
- The local trees contained in the simulated data. This file is a list of Newick trees each of which is preceded by the number of sites that share the current local tree.
- A full description of the graph representing the ancestry of the sample in the DOT language (Fig. 2 in the Main Text). This can be used in conjunction with the graphviz and the DOT program to produce figures illustrating the ancestry. The examples show the ancestry with and without the ancestral material included at each node. The clonal genealogy is shown in bold and external recombination events are shown in red. In the graph showing the ancestral material, the ancestral material remaining at each node is shown in grey and any external genetic material is shown in red.

Examples

To simulate 100 genomes each 1Mbp long with an internal recombination rate $R_i = 0.01$ and mutation rate $\theta = 0.01$ run:
./SimBac -N 100 -B 1000000 -R 0.01 -T 0.01 -o sequences.fasta -c clonal.nwk -l local.nwk
This produces the simulated sequences and the clonal genealogy in the files 'sequences.fasta' and 'clonal.nwk'. The local trees are written to 'local.nwk'

To simulate 100 genomes with internal and external recombination rate $R_e = 0.01$ and average break length of 500bp run:
./SimBac -N 100 -B 1000000 -R 0.01 -D 500 -r 0.01 -e 500 -b internal.log -f external.log
This produces two log files with the start- and end-points of all internal and external recombination events.

To simulate sequences undergoing internal and external recombination with mutation in an external recombinant interval occurring with probability in the interval $[0.5, 1]$, run:
./SimBac -N 100 -B 100000 -R 0.01 -D 500 -r 0.01 -e 500 -m 0.5 -M 1 -o sequences.fasta -c clonal.nwk
This produces the sequences and clonal genealogy.

To produce a DOT file with the ancestral information included in the graph run:
./SimBac -n 10 -B 1000 -R 0.01 -D 50 -r 10 -e 50 -d graph.dot -a

To simulate a linear genome, add a large gap to the end of the genome to prevent any recombinant intervals including both the first and last elements of the genome. For example to simulate a linear genome of length 100kbp run:
./SimBac -N 100 -B 100000 -G 1000000 -o sequences.fasta -c clonal.nwk
This places a gap of 1Mbp at the end of the genome.

Supplementary Methods

Hereby we will use the notation of [2], except that we will assume that there is a circular genome of length G (with sites $1 \dots G$), and that ancestral material of each lineage is a subset of this genome. Ancestral material for a sample (an ARG tip) need not be the whole genome, but might be a subset of the genome made of different loci, for example in the case of MLST data, so that we can simulate both genome data or MLST data. Ancestral material for a node consists of b non-overlapping ordered intervals, $I_1 \dots I_b$, of lengths respectively $L_1 \dots L_b$, and with $I_i = [s_i, e_i]$ (implying $e_i - s_i = L_i - 1$). Also for easiness of presentation (due to genome circularity) we will set $e_0 = e_b - G$, which is intendedly negative. The recombination rate per site per genome will be $R/2$. It should be noted that $\rho = 2R$, where ρ is the rate of recombination initiation or termination in LDhat [3]. Lastly, recombining intervals have a geometric distribution with mean δ.

Effective recombination rate for a lineage

In [2], the recombination rate per site is $R/2$. We call a the ancestral material of this lineage. If a recombination event happens on the considered lineage, then a recombining interval r is picked at random from the genome, and if $r \cap a \neq \emptyset$ (and $a - r \neq \emptyset$ for lineages not in the clonal frame) then the two new recombining lineages are created, otherwise the recombination event is rejected.

Here we propose to sample recombination events and recombining intervals conditional on $r \cap a \neq \emptyset, a - r \neq \emptyset$, or just on $r \cap a \neq \emptyset$ for lineages in the clonal frame, such that no rejection ever occurs while simulating. To do this, we first define a lineage-specific effective recombination rate. This is the rate at which recombination events occur satisfying $r \cap a \neq \emptyset, a - r \neq \emptyset$ (or just $r \cap a \neq \emptyset$ for clonal frame lineages). As in [2], we assume that the rate of initiation of a recombination event is the same for each site of the genome. Under these assumptions, and assuming as in [1] a geometric distribution with mean δ for recombination interval lengths, the rate at which a recombination event is started between e_0 and s_1, and includes s_1, is:

$$\frac{R_{s_1-e_0}}{2} = \frac{R}{2}\sum_{i=0}^{s_1-e_0-1}(1-\delta^{-1})^i =$$
$$= \frac{R}{2}\left[\sum_{i=0}^{\infty}(1-\delta^{-1})^i - \sum_{i=s_1-e_0}^{\infty}(1-\delta^{-1})^i\right] =$$
$$= \frac{R}{2}\left[\delta - \delta(1-\delta^{-1})^{(s_1-e_0)}\right] =$$
$$= \frac{R}{2}\delta(1-(1-\delta^{-1})^{(s_1-e_0)}).$$

Where $\left(1-\delta^{-1}\right)^i$ is the probability of a recombinant break having length greater than i. Now, let us assume we have a lineage with ancestral material $a = \cup_{i=1}^{b}[s_i, e_i]$ union of non-empty, ordered, disjoint intervals. As mentioned before, $e_0 = e_b - G$. The amount of ancestral material in a lineage is defined as: $L = \sum_{i=1}^{b} L_i$ The rate of recombination events satisfying $r \cap a \neq \emptyset$ for that lineage is then:

$$\frac{R_a}{2} = \left(\sum_{i=1}^{b}\frac{R_{s_i-e_{i-1}}}{2}\right) + \frac{R}{2}(L-b).$$

Finally, the lineage-specific recombination rate satisfying $r \cap a \neq \emptyset$, and $a - r \neq \emptyset$ is:

$$\frac{R'_a}{2} = \frac{R_a}{2} - \left(\sum_{i=1}^{b}\frac{R_{s_i-e_{i-1}}}{2}(1-\delta^{-1})^{G-(s_i-e_{i-1})}\right) - \frac{R}{2}(1-\delta^{-1})^{G-1}(L-b).$$

Additionally, for a clonal lineage without ancestral material the recombination rates will be 0.

Probability of recombination initiating sites

Conditional on an effective recombination event on a non clonal frame lineage (that is, satisfying $r \cap a \neq \emptyset, a - r \neq \emptyset$) occurring on ancestral material a, the probability that the first ancestral site affected by r is s_i is:

$$P'_{s_i} = \frac{R_{s_i - e_{i-1}}(1 - (1 - \delta^{-1})^{G-(s_i - e_{i-1})})}{R'_a},$$

and the probability that it is any other site in a is

$$\frac{R(1 - (1 - \delta^{-1})^{G-1})}{R'_a}.$$

If the considered recombining lineage is in the clonal frame instead (with recombination satisfying only $r \cap a \neq \emptyset$), the probabilities are

$$P_{s_i} = \frac{R_{s_i - e_{i-1}}}{R_a},$$

and

$$\frac{R}{R_a}$$

respectively.

After the starting site of $r \cap a$ has been picked, the ending site of r is chosen according to a geometric distribution with mean δ for a lineage in the clonal frame. In a non-clonal lineage, the ending site of r is chosen according to the same geometric distribution, but conditional on $|r| \leq G - (s_i - e_{i-1})$ if the starting site of r is s_i, or $|r| \leq G - 1$ otherwise.

External recombination events

Simulation of external recombination events follows the same protocol as that of a clonal recombination event, with $R_e/2$ and δ_e replacing $R/2$ and δ, respectively. As we are only interested in the imported fragment from the external species, the recombinant interval need only satisfy the condition $r \cap a \neq \emptyset$, as in a clonal lineage.

Technical description of SimBac

Here, n denotes the number of isolates for which the data is to be simulated. For a genome of length G, simulate the ARG with internal recombination rate $R_i/2$ and external recombination rate $R_e/2$. The average length of internal and external recombining segments are given by δ_i and δ_e, respectively.

Input: $n,\ R_i/2,\ R_e/2,\ \delta_i,\ \delta e,\ G$
Output: Simulated ancestral recombination graph with clonal genealogy

Set number of lineages $k = n$
for $x = 1 \ldots n$ **do**
  Calculate internal and external recombination rates, $\frac{R_{i,x}}{2}$, $\frac{R_{e,x}}{2}$
  Ancestral material a_x is given by $[0, G]$
  Lineage x is clonal
end for
while $k > 1$ **do**
  Calculate the rates of internal and external recombination given by:
  $\frac{R_i}{2} = \sum_{x=1}^{k} \frac{R_{i,x}}{2}$ and $\frac{R_e}{2} = \sum_{x=1}^{k} \frac{R_{e,x}}{2}$
  Increment current time by an amount distributed exponentially with parameter $\binom{k}{2} + \frac{R_i}{2} + \frac{R_e}{2}$
  Let $u \sim U(0,1)$
  if $u < k(k-1)/(k(k-1) + R_i + R_e)$ **then**
    Coalescent event
    Choose two lineages x and y at random from the k remaining nodes and replace with the new lineage z
    The lineage z is clonal if x or y is clonal
    The ancestral material of z is $a_z = a_x \cup a_y$
    Update total number of lineages containing each element of the genome
    for all Nucleotides **do**
      if Number of lineages containing given nucleotide is one **then**
        Remove nucleotide from a_z
      end if
    end for
    Calculate the internal and external rate of recombination for the new lineage, $\frac{R_{i,z}}{2}$ and $\frac{R_{e,z}}{2}$
    The number of lineages, k is decreased by one
  else if $u < (k(k-1) + R_i)/(k(k-1) + R_i + R_e)$ **then**
    Internal recombination event
    Choose one lineage x weighted by $\frac{R_{i,x}}{2}$
    Determine a recombining interval, r, distributed geometrically with parameter δ_i.
    if x is clonal **then**
      Choose r such that $r \cap a_x \neq \emptyset$
    else
      Choose r such that $r \cap a_x \neq \emptyset$ and $a - r \neq \emptyset$
    end if
    Create two new lineages, y and z.
    Lineage z is clonal if x is clonal
    $a_y = a_x \cap r$ and $a_z = a_x - r$
    Lineage y is not clonal
    Calculate the new recombination rates for lineages y and z, $\frac{R_{i,y}}{2}$, $\frac{R_{e,y}}{2}$, $\frac{R_{i,z}}{2}$ and $\frac{R_{e,z}}{2}$
    The number of lineages, k is increased by one
  else
    External recombination event
    Choose one lineage, x weighted by $\frac{R_{e,x}}{2}$
    Choose a recombinant interval, r, distributed geometrically with parameter δ_e conditioned on $r \cap a_x \neq \emptyset$
    The material satisfying $a_x \cap r$ will be simulated as genetic material from an external species
  end if
end while

Methods Validation

Fig. S 1: Validation of SimBac. To validate SimBac we compared summary statistics of its simulated data to those of simMLST and ms. Not all statistics are available for every software. Genome length is 10^5bp. On X axis is always shown the scaled, per-site recombination rate R and error bars represent $\pm$ 1 standard deviations. 10 simulations were performed for R=0.02 and 0.05 in simMLST in the top two plots, 100 simulations in all other cases. (a) Total number of recombination events. (b) Height of the ARG. (c) Number of local trees (identical neighbouring local trees were merged). (d) Average sum of branch lengths for local trees (ms values are scaled by a factor of 2 as it assumes diploidy, while SimBac and SimMLST assume haploidy).

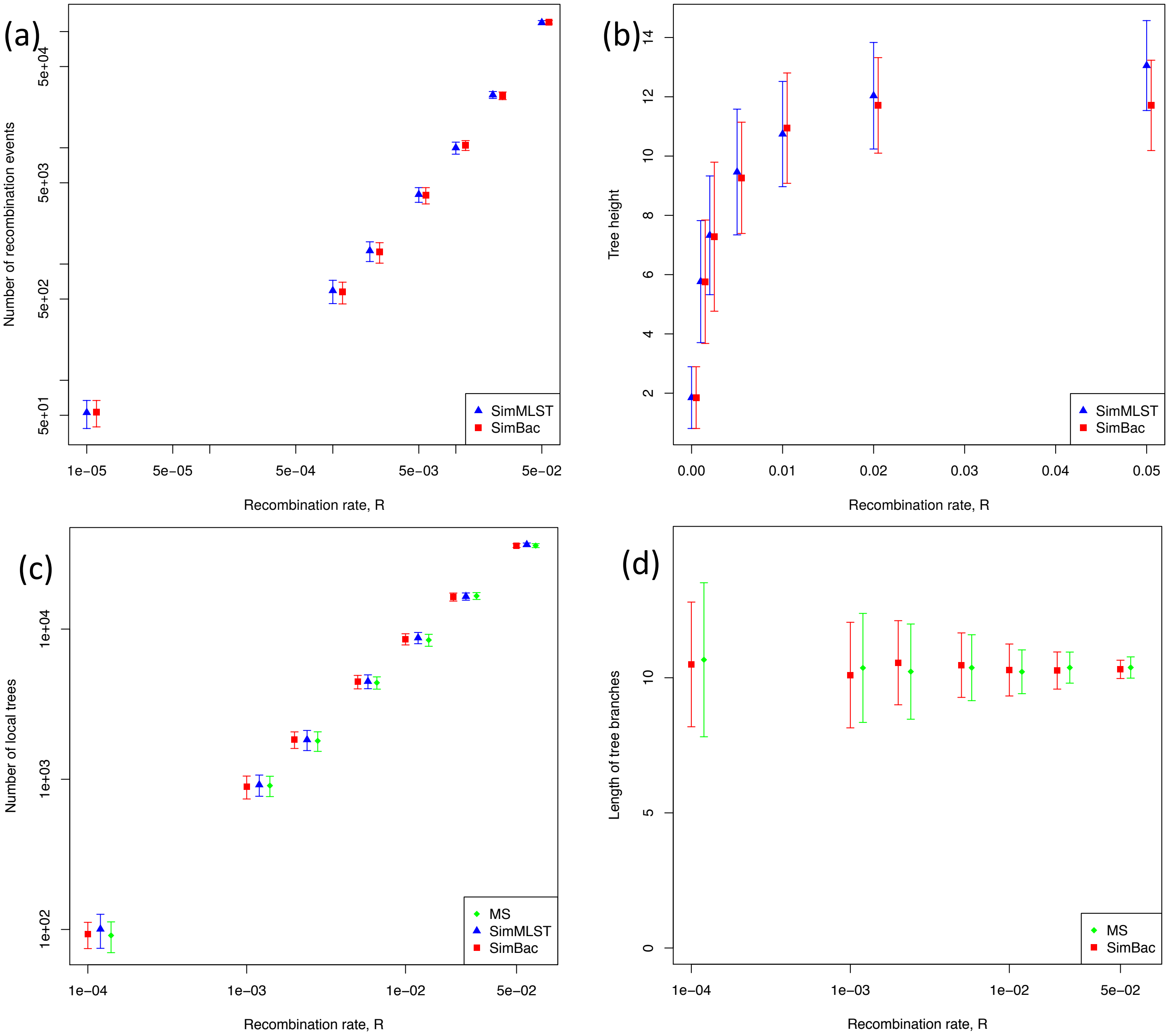

References

[1] Didelot,X., Falush,D. (2006) Inference of Bacterial Microevolution Using Multilocus Sequence Data. *Genetics*, **175**, 1251-1266.

[2] Didelot, X. Lawson, D, Falush, D. (2009) SimMLST: simulation of multi-locus sequence typing data under a neutral model *Bioinformatics*, **25**(11): 1442–1444.

[3] McVean, G. Awadalla, P. Fearnhead, P. (2002) A Coalescent-Based Method for Detecting and Estimating Recombination From Gene Sequences *Genetics*, **160**: 1231–1241.
